# *KRAS* overexpression independent of *RAS* mutations confers an adverse prognosis in cytogenetically normal acute myeloid leukemia

**DOI:** 10.18632/oncotarget.19798

**Published:** 2017-08-02

**Authors:** Jing-Dong Zhou, Dong-Ming Yao, Xi-Xi Li, Ting-Juan Zhang, Wei Zhang, Ji-Chun Ma, Hong Guo, Zhao-Qun Deng, Jiang Lin, Jun Qian

**Affiliations:** ^1^ Department of Hematology, Affiliated People’s Hospital of Jiangsu University, Zhenjiang, Jiangsu, People’s Republic of China; ^2^ Laboratory Center, Affiliated People’s Hospital of Jiangsu University, Zhenjiang, Jiangsu, People’s Republic of China; ^3^ The Key Laboratory of Precision Diagnosis and Treatment of Zhenjiang City, Zhenjiang, Jiangsu, People’s Republic of China

**Keywords:** *RAS*, expression, mutation, prognosis, acute myeloid leukemia

## Abstract

The prognostic value of *RAS* mutations has been systematically investigated in acute myeloid leukemia (AML). However, clinical significance of *RAS* expressions in AML remains poorly determined. To explore the clinical significance, we analyzed *KRAS* and *NRAS* expressions in 143 *de novo* AML patients by real-time quantitative PCR. *KRAS* and *NRAS* expressions were significantly up-regulated in AML patients. *KRAS* and *NRAS* mutations were identified in 4% (6/143) and 8% (12/143) of these patients, respectively. However, no significant association was observed between *RAS* mutations and expressions. High *KRAS* expression was associated with older age, higher white blood cells, and a tendency of higher platelets, whereas high *NRAS* expression was only correlated with older age. Complete remission (CR) rate and overall survival of AML patients were adversely affected by *KRAS* overexpression, but not *NRAS* overexpression. Multivariate analysis revealed that *KRAS* acted as an independent prognostic predictor in cytogenetically normal AML (CN-AML). Moreover, the prognostic value of *KRAS* expression was validated using the published data from Gene Expression Omnibus datasets. In the follow-up patients, *KRAS* expression rather than *NRAS* expression in CR time tended to decrease compared to newly diagnosis time, and both *KRAS* and *NRAS* expressions were significantly increased when in relapse time. Our findings revealed that *RAS* overexpression and mutations were common events in AML with potential therapeutic target value. *KRAS* overexpression independent of *RAS* mutations conferred an adverse prognosis in CN-AML.

## INTRODUCTION

Acute myeloid leukemia (AML) is an aggressive hematopoietic malignancy associated with severe morbidity and poor clinical outcome [1, 2]. Cytogenetic abnormalities of AML assessed at diagnosis are generally recognized as the most valuable independent prognostic factors in AML, allowing the classification of AML into favorable, intermediate, and poor prognostic groups [2, 3]. However, in approximately 50% of AML patients, no cytogenetic abnormality is detectable at the diagnosis time, often called as cytogenetically normal AML (CN-AML) [1, 2]. Such patients are in an intermediate-risk prognostic category, but among them are subgroups of patients who have molecular markers associated with either a favorable prognosis or an unfavorable prognosis [2–4]. Over the past decades, several gene mutations, such as *CEBPA*, *NPM1*, *FLT3*-ITD, *C-KIT*, *DNMT3A*, and *IDH1/2*, and changes in gene expression, such as overexpression of *BAALC*, *ERG*, *EVI1*, and *MN1*, have been discovered to strongly affect clinical outcome of CN-AML patients [4]. Accordingly, further refinement of relevant molecular alterations in different AML subgroups might eventually result in more individual treatment approaches and potentially improve outcome.

*RAS* proto-oncogenes, including *KRAS*, *NRAS*, and *HRAS*, encode a membrane-localized G protein of 21 kDa regulate the growth and differentiation of many cell types [5–7]. RAS proteins are located on the inner surface of the plasma membrane and act as molecular switches that transduce extracellular signals to the nucleus [5–7]. It is inactive when bound to GDP and active when bound to GTP [5–7]. *RAS* activation caused by its mutation giving rise to an abnormal protein resistant to GTP hydrolysis by GTPase leads to a constitutively active GTP-bound protein that stimulates a critical network of signal transduction pathways that result in cellular proliferation, survival, and differentiation [5–7]. *RAS* mutations at codons 12, 13, and 61 are common events in human cancers, and are frequently detected in AML with their clinical relevance been systematically determined [5–7]. Herein, we investigated *RAS* expressions and their clinical significances in *de novo* AML patients.

## RESULTS

### RAS expressions and mutations in AML

**Figure 1:**
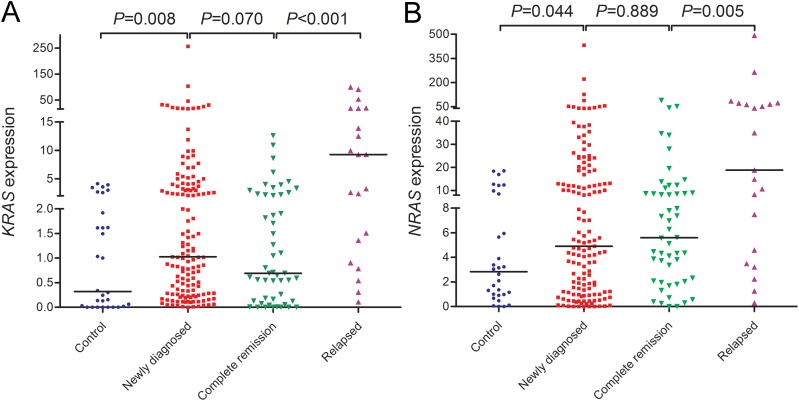
*RAS* expressions in controls and AML patients including newly diagnosed AML, AML at complete remission time, and relapsed AML **(A)** *KRAS* expression. **(B)** *NRAS* expression.

We first examined *KRAS* and *NRAS* expressions in controls and newly diagnosed AML patients. *KRAS* expression in AML patients (median 1.024) was significantly up-regulated than controls (median 0.319) (*P*=0.008, Figure 1A). By the cut-off value (defined as mean+2SD in controls), *KRAS* overexpression was identified in 35/143 (24%) of AML patients. Moreover, increased *NRAS* expression was also found in AML patients compared with controls (median 4.896 vs 2.838) (*P*=0.044, Figure 1B), and identified in 37/143 (26%) of AML patients based on the cut-off value (defined as mean+2SD in controls). Moreover, *KRAS* expression was positively correlated with *NRAS* expression in AML patients (R=0.605, *P*<0.001).

*KRAS* and *NRAS* mutations were scanned in all patients. *KRAS* mutation was found in 4% (6/143) patients, whereas 8% (12/143) patients harbored *NRAS* mutation. Notably, no significant differences was observed regarding *KRAS* (median 2.759 vs 1.017, *P*=0.330) and *NRAS* (median 16.148 vs 4.738, *P*=0.355) expression in patients with and without *KRAS* mutation. In addition, patients with and without *NRAS* mutation also showed similar level of *KRAS* (median 1.274 vs 1.017, *P*=0.350) and *NRAS* (median 5.055 vs 4.896, *P*=0.884) expression. All these results suggested that *RAS* expressions were not correlated with *RAS* mutations in AML.

### Clinical and laboratory characteristics of AML

Previous study has revealed the clinical significance of *RAS* mutations in *de novo* AML patients [8]. Herein, we further investigated the correlation of *RAS* expressions with clinico-pathologic features. As is shown in Table 1, *KRAS* high-expressed (*KRAS*^high^) patients were associated with older age (*P*=0.034), higher white blood cells (*P*=0.007), and a tendency of higher platelets (*P*=0.057), whereas *NRAS* high-expressed (*NRAS*^high^) patients were only correlated with older age (*P*=0.009). Additionally, only *KRAS* but not *NRAS* expression showed significant differences in the distribution of karyotypes, and *KRAS* overexpression showed lower frequency in t(15;17) subtypes (*P*=0.048). Among gene mutations, *KRAS* overexpression was correlated with *U2AF1* mutation (*P*=0.033), whereas *NRAS* overexpression might be associated with *IDH1/2* mutations (*P*=0.059).

**Table 1:** Correlation analyses of *KRAS* and *NRAS* expressions with clinic-pathologic features in AML patients

Patient’s parameters	*KRAS* expression			*NRAS* expression		
	Low (n=108)	High (n=35)	*P*	Low (n=106)	High (n=37)	*P*
Sex, male/female	62/46	25/10	0.166	64/42	23/14	1.000
Age, median (range)	54 (15-87)	60 (10-93)	0.034	54 (10-93)	63 (17-87)	0.009
WBC, median (range)	13.2 (0.3-528.0)	34.5 (0.8-197.7)	0.007	16.6 (0.3-528.0)	23.0 (1.2-135.4)	0.543
HB, median (range)	75 (34-144)	82 (34-135)	0.844	77 (34-138)	76 (34-144)	0.863
PLT, median (range)	32 (3-415)	43 (9-399)	0.057	32 (3-415)	46 (9-399)	0.181
BM blasts, median (range)	47.0 (1.0*-97.5)	38.8 (21.5-92.0)	0.983	44.8 (1.0*-97.5)	48.5 (6.5*-92.0)	0.855
Karyotypic classifications			0.230			0.399
Favorable	34 (31.5%)	6 (17.1%)		31 (29.2%)	8 (21.6%)	
Intermediate	60 (55.6%)	23 (65.7%)		62 (58.5%)	21 (56.8%)	
Poor	12 (11.1%)	4 (11.4%)		11 (10.4%)	6 (16.2%)	
No data	2 (1.9%)	2 (5.7%)		2 (1.9%)	2 (5.4%)	
Karyotypes			0.048			0.250
Normal	46 (42.6%)	16 (45.7%)		46 (43.4%)	16 (43.2%)	
t(8;21)	10 (9.3%)	3 (8.6%)		8 (7.5%)	5 (13.5%)	
inv(16)	0 (0%)	1 (2.9%)		1 (0.9%)	0 (0%)	
t(15;17)	24 (22.2%)	1 (2.9%)		22 (20.8%)	3 (8.1%)	
+8	3 (2.8%)	2 (5.7%)		5 (4.7%)	0 (0%)	
t(9;22)	0 (0%)	1 (2.9%)		0 (0%)	1 (2.7%)	
-5/5q-	2 (1.9%)	0 (0%)		1 (0.9%)	1 (2.7%)	
-7/7q-	1 (0.9%)	0 (0%)		1 (0.9%)	0 (0%)	
Complex	10 (9.3%)	4 (11.4%)		9 (8.5%)	5 (13.5%)	
Others	10 (9.3%)	5 (14.3%)		11 (10.4%)	4 (10.8%)	
No data	2 (1.9%)	2 (5.7%)		2 (1.9%)	2 (5.4%)	
Gene mutations						
*KRAS* (+/-)	5/103	1/34	1.000	3/103	3/34	0.339
*NRAS* (+/-)	7/101	5/30	0.167	9/97	3/34	1.000
Double *CEBPA* (+/-)	4/88	0/26	0.575	4/85	0/29	0.571
*NPM1* (+/-)	8/84	3/23	0.705	7/82	4/25	0.461
*FLT3*-ITD (+/-)	12/80	2/24	0.526	11/78	3/26	1.000
*C-KIT* (+/-)	5/87	1/25	1.000	4/85	2/27	0.635
*IDH1* (+/-)	1/91	1/25	0.394	0/89	2/27	0.059
*IDH2* (+/-)	1/91	1/25	0.394	0/89	2/27	0.059
*DNMT3A* (+/-)	5/87	3/23	0.372	7/82	1/28	0.677
*U2AF1* (+/-)	1/91	3/23	0.033	3/86	1/28	1.000
*SRSF2* (+/-)	2/90	1/25	0.530	2/87	1/28	1.000
CR (+/-)	51/49 (51.0%)	11/24 (31.4%)	0.051	48/50 (49.0%)	14/23 (37.8%)	0.333

AML, acute myeloid leukemia; WBC, white blood cells; HB, hemoglobin; PLT, platelets; BM, bone marrow; CR, complete remission. *, AML patients less than 20% BM blasts often with typical cytogenetics such as t(15;17).

### Prognostic value of RAS expressions and mutations in AML

Follow-up data was available in 135 AML patients after receiving induction chemotherapy (median: 10 months, 95% CI: 6.374-123.626). In whole-cohort AML, *KRAS*^high^ patients showed had an obvious tendency of lower complete remission (CR) rate, whereas *NRAS* did not (Table 1). Both *KRAS* and *NRAS* expressions were not correlated with CR rate among non-acute promyelocytic leukemia (APL) patients [43% (34/79, *KAS*^low^) vs 29% (10/34, *KRAS*^low^), *P*=0.210 and 41% (32/79, *NRAS*^low^) vs 35% (12/34, *NRAS*^high^), *P*=0.677]. Among CN-AML, *KRAS*^high^ and *NRAS*^high^ patients also presented an obvious tendency of lower CR rate [47% (20/43, *KRAS*^low^) vs 25% (4/16, *KRAS*^high^), *P*=0.078 and 47% (20/43, *NRAS*^low^) vs 25% (4/16, *NRAS*^high^), *P*=0.078].

**Figure 2:**
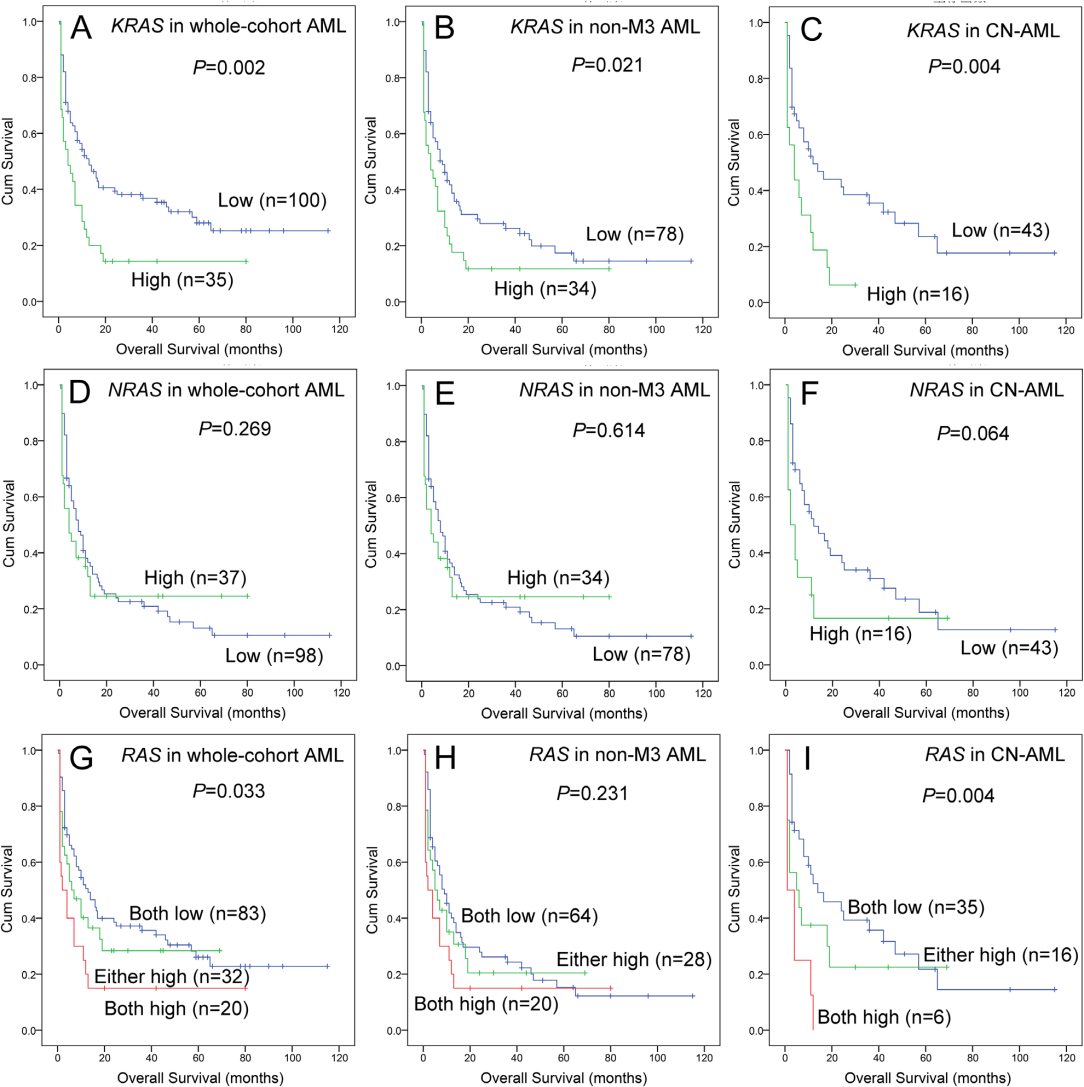
The impact of *RAS* expressions on overall survival in AML patients **(A, B** and **C)** For *KRAS* expression in whole-cohort AML, non-APL AML, and CN-AML patients. **(D, E** and **F)** For *NRAS* expression in whole-cohort AML, non-APL AML, and CN-AML patients. **(G, H** and **I)** For *RAS* expression in whole-cohort AML, non-APL AML, and CN-AML patients, “both low” indicated neither *KRAS* nor *NRAS* overexpression, “either high” indicated either *KRAS* or *NRAS* overexpression, “both high” indicated both *KRAS* and *NRAS* overexpression.

Survival analyses were further performed in 135 AML patients. Kaplan-Meier analyses demonstrated that *KRAS*^high^ was associated with shorter overall survival (OS) time among whole-cohort AML, non-APL AML, and CN-AML patients (Figure 2A, 2B and 2C). However, *NRAS*^high^ was not correlated with OS time in whole-cohort AML and non-APL AML (Figure 2D and 2E), but had an obvious tendency of shorter OS time in CN-AML (Figure 2F). Next, we classified patients into three groups (both low vs either high vs both high) regarding *RAS* expressions, and showed in Figure 2G, 2H and 2I (whole-cohort AML, non-APL AML, and CN-AML). Cox regression analyses were further performed to determine the prognostic impact of *RAS* expressions in AML, and showed that *KRAS* was an independent prognostic biomarker in CN-AML (Table 2) but not in whole-cohort AML and non-APL AML patients (data not shown).

**Table 2:** Univariate and multivariate analyses of prognostic factors for overall survival in cytogenetically normal AML patients

	Univariate analysis		Multivariate analysis	
	Hazard ratio (95% CI)	*P* value	Hazard ratio (95% CI)	*P* value
*KRAS* expression	2.443 (1.277-4.675)	0.007	2.464 (1.113-5.453)	0.026
*NRAS* expression	1.798 (0.937-3.452)	0.078	1.805 (0.467-2.522)	0.849
Age	2.125 (1.166-3.874)	0.014	1.812 (0.895-3.670)	0.099
WBC	2.618 (1.431-4.788)	0.002	1.985 (1.001-3.938)	0.050
CEBPA* mutations	1.013 (0.241-4.262)	0.986		
*NPM1* mutations	0.679 (0.240-1.927)	0.467		
*FLT3*-ITD mutations	0.607 (0.211-1.742)	0.353		
*C-KIT* mutations	0.734 (0.100-5.402)	0.761		
*KRAS* mutations	5.323 (1.534-18.476)	0.008	8.702 (1.794-42.209)	0.007
*NRAS* mutations	1.609 (0.673-3.850)	0.285		
*IDH1* mutations	8.565 (1.759-41.710)	0.008	6.609 (1.279-34.135)	0.024
*IDH2* mutations	5.707 (0.714-45.644)	0.101		
*DNMT3A* mutations	1.412 (0.545-3.656)	0.477		
*U2AF1* mutations	2.179 (0.290-16.342)	0.449		
*SRSF2* mutations	2.915 (0.669-12.705)	0.154		

WBC, white blood cells. Variables including age (≤60 vs. >60 years), WBC (≥30×10^9^ vs. <30×10^9^ /L), *RAS* expression (lower vs. higher), and gene mutations (mutant vs. wild-type). Multivariate analysis includes variables with *P*<0.100 in univariate analysis. *, double *CEBPA* mutations.

**Figure 3:**
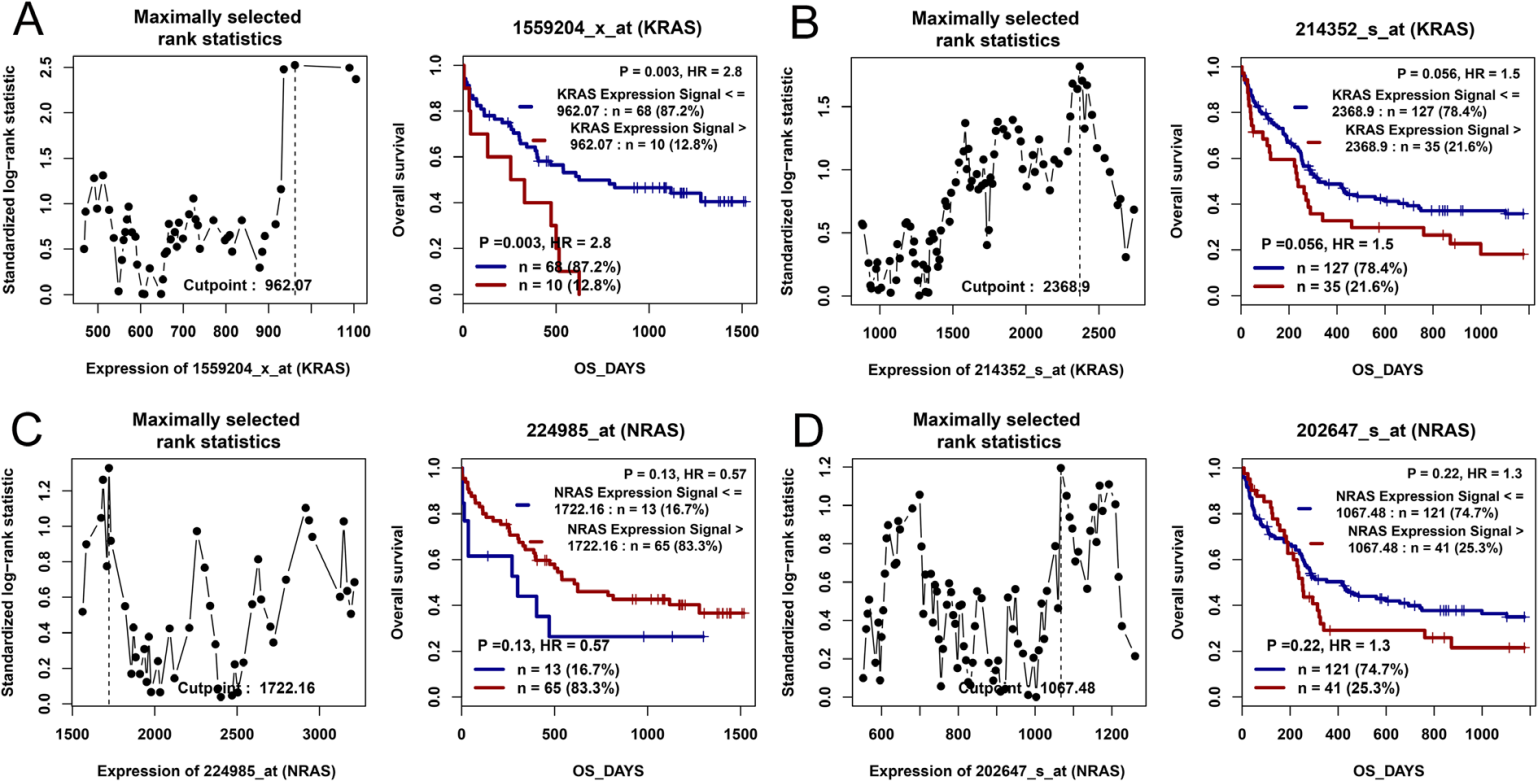
The impact of *RAS* expressions on overall survival in CN-AML patients by bioinformatics analysis Two independent cohorts of 78 and 162 CN-AML patients were obtained from Gene Expression Omnibus data (http://www.ncbi.nlm.nih.gov/geo/; accession number GSE12417). Survival analysis was performed through the online web tool Genomicscape (http://genomicscape.com/microarray/survival.php). **(A)** For *KRAS* in a cohort of 78 CN-AML patients. **(B)** For *KRAS* in a cohort of 162 CN-AML patients. **(C)** For *NRAS* in a cohort of 78 CN-AML patients. **(D)** For *NRAS* in a cohort of 162 CN-AML patients.

The published data from two independent cohorts of CN-AML patients available in Gene Expression Omnibus (GEO) databases were set as the independent validation cohort. Through the online tools GenomicScape, *KRAS* overexpression was significantly related to shorter OS time (Figure 3A and 3B), whereas *NRAS* was not found (Figure 3C and 3D).

**Figure 4:**
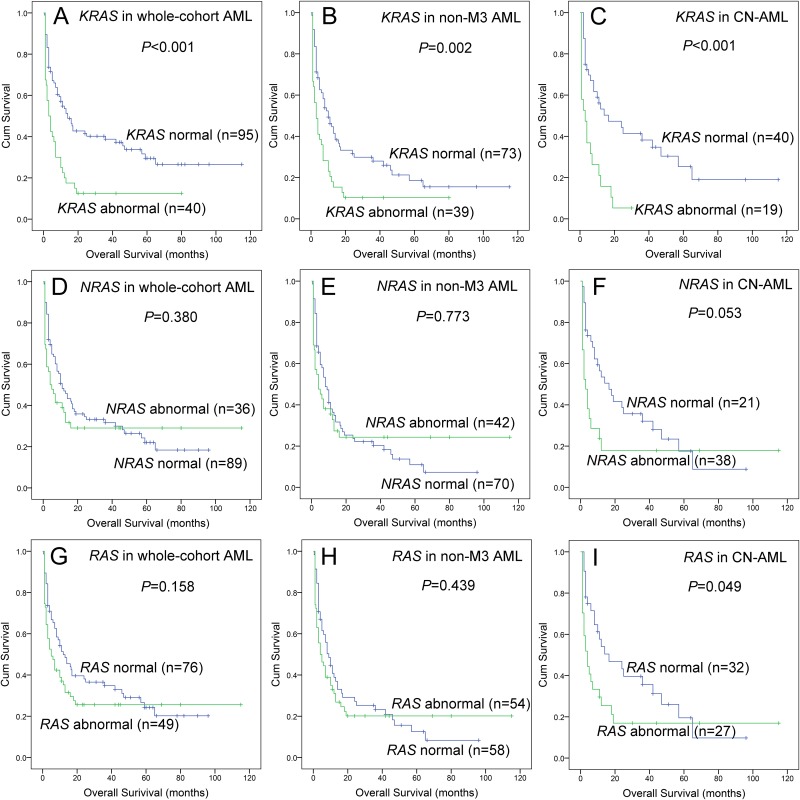
The impact of *RAS* abnormalities on overall survival in AML patients **(A, B** and **C)** For *KRAS* abnormalities in whole-cohort AML, non-APL AML, and CN-AML patients. **(D, E** and **F)** For *NRAS* abnormalities in whole-cohort AML, non-APL AML, and CN-AML patients. **(G, H** and **I)** For *RAS* abnormalities in whole-cohort AML, non-APL AML, and CN-AML patients. “Abnormal” indicated gene with overexpression or mutation, whereas “normal” indicated gene without overexpression and mutation.

Lastly, we further analyzed the impact of *RAS* abnormalities (overexpression and mutation) on prognosis. We divided patients into groups regarding *RAS* expressions and mutations including *KRAS* normal (without mutation and overexpression) vs *KRAS* abnormal (with mutation or overexpression), *NRAS* normal vs *NRAS* abnormal, and *RAS* normal vs *RAS* abnormal. All the results were presented in Figure 4.

### RAS expressions in the surveillance of AML

To observe the dynamic change of *RAS* expressions in AML patients of different clinical stages, we further detected *RAS* expressions in 51 patients who achieved CR after induction therapy and 21 relapsed patients. As was shown in Figure 1A and 1B, *KRAS* expression rather than *NRAS* expression in CR time tended to decrease compared to newly diagnosis time, and both *KRAS* and *NRAS* expressions were significantly increased when in relapse time.

## DISCUSSION

The signal switch molecules RAS proteins play crucial roles in relaying growth-promoting signals from cell surface receptors [5–7]. Oncogenic point mutations of *RAS* are identified in -30% of human cancers especially in pancreatic cancer, lung cancer, and colon cancer [5–7]. Significantly, *RAS* mutations, usually in *KRAS* and *NRAS*, are also frequent events in myeloid malignancies, and have been detected in 3% to 40% of myelodysplastic syndrome (MDS) and AML [5–7]. In this study, *KRAS* and *NRAS* mutations were identified in 4% and 8% AML patients, which showed lower percentage compared to previous study. Possible reasons were that we only detected hot-spot mutations in *RAS*, or the differences in ethnics. Moreover, the prognostic value of *RAS* mutations in AML remains controversial. Several investigators reported *RAS* mutations emerged as significant predictors for improved clinical outcome [9]. Meanwhile, *NRAS* mutation was associated with adverse prognosis and increased risk of leukemia transformation in MDS [10]. Conversely, the opponents hold the view that *RAS* mutations were associated with distinct cytogenetic subgroups, usually M4, but not correlated with prognosis in AML patients [11, 12]. Our previous study also analyzed the clinical significance of *RAS* mutations in *de novo* AML, but did not have an independent effect on prognosis [8]. In addition, we also did not observe the impact of *FLT3*, *NPM1* and *CEBPA* mutations on prognosis, which might be due to the small size of patients with mutations in our cohort.

In the present study, we mainly focused on *RAS* expressions in AML, and found that both *KRAS* and *NRAS* overexpression were common events more frequently than *RAS* mutations in *de novo* AML patients. Notably, our study did not find significant association of *RAS* overexpression with *RAS* mutations. Similarly, a recent report also showed *KRAS* mutation did not correlate with mRNA expression [13]. These results suggested that *RAS* overexpression and mutations were independent events contributing to the pathogenesis of AML. Moreover, *RAS* overexpression rather than *RAS* mutations might play more critical roles in leukemogenesis. The underlying mechanism during leukemogenesis caused by *RAS* overexpression needed further studies. Interestingly, we observed that *KRAS* overexpression was associated with *U2AF1* mutations, whereas *NRAS* overexpression was associated with *IDH1/2* mutations. As is well known, RNA splicing factor gene *U2AF1* and isocitrate dehydrogenase gene *IDH1/2* mutations are recurrent in *de novo* AML especially in CN-AML, and have a prognostic impact on assessing treatment outcome [14, 15]. However, it was the first time to report the association of *RAS* overexpression with these gene mutations. No functional studies were found to verify our results. In addition, due to the limited cases of *RAS* mutations in our cohort, perspective studies in a larger cohort of AML patients are required to confirm these findings, and further reveal the underlying molecular mechanism.

Importantly, our study revealed the impact of *RAS* expressions on clinical outcome of AML. Firstly, a negative effect of *KRAS* overexpression was observed among whole-cohort AML and CN-AML patients. The results indicated that *KRAS* activation may be associated with chemoresistance in the induction therapy of AML. Although there were no functional studies to validate the hypothesis in AML, several investigations revealed the oncogenic role of *KRAS* abnormalities (overexpression or mutation) were associated with resistant to anticancer drug treatments phenomenally and/or mechanically [16–18]. Secondly, both our data and the published GEO databases showed that *KRAS* overexpression was a prognostically adverse predictor in CN-AML patients. More importantly, *KRAS* expression may increase the power in predicting prognosis when combined with other molecular alterations such as *NRAS* expression or *NRAS/KRAS* mutations. The prognostic value of *KRAS* expression has been determined in various cancers. For instance, Chen et al disclosed that *KRAS* overexpression predicted poor prognosis in patients with colorectal cancer [19]. Moreover, *RAS* expressions as an independent indicator of patient outcomes in lung cancer treated with bevacizumab plus chemotherapy [20]. Thirdly, we further found *RAS* expressions could be used as a biomarker for monitoring disease treatment and recurrence in AML. Collectively, these above results emphasized a more crucial role of *KRAS* from *RAS* family in the process of leukemogenesis, and could act as a potential therapeutic target for designing cancer gene therapy.

Taken together, our findings reveal that *RAS* overexpression and mutations are common events in AML with potential therapeutic target value. *KRAS* overexpression independent of *RAS* mutations confers an adverse prognosis in CN-AML.

## MATERIALS AND METHODS

### Patients and samples

This study was approved by the Institutional Review Board of the Affiliated People’s Hospital of Jiangsu University. After written informed consents were obtained from all participants, bone marrow (BM) was collected from 143 *de novo* AML patients at newly diagnosis time, 51 AML patients at CR time, and 21 AML patients at relapse time. The diagnosis and classification of AML patients were established according to the 2008 World Health Organization (WHO) criteria. BM samples from 30 healthy donors were collected as controls. The separation of BM mononuclear cells (BMMNCs) was performed using Lymphocyte Separation Medium (TBD Sciences, Tianjin, China) and washed twice with PBS.

### Treatment regimen

All the AML patients received chemotherapy including induction therapy and subsequent consolidation treatment as reported in our previous literature [21, 22]. For patients with APL, induction therapy was oral all-trans retinoic acid (ATRA) together with daunorubicin in combination with cytarabine, and maintenance therapy was oral mercaptopurine, oral methotrexate, and oral ATRA over two years. For non-APL patients, induction therapy was one or two courses of daunorubicin combined with cytarabine, whereas subsequent consolidation treatment included high-dose cytarabine, mitoxantrone combined with cytarabine, homoharringtonine together with cytarabine, and etoposide in combination with cytarabine.

### Cytogenetic analyses

Karyotypes were analyzed at the newly diagnosis time by conventional R-banding method and karyotype risk was classified according to reported previously [23].

### RNA isolation and reverse transcription

Total RNA was extracted from the BMMNCs using Trizol reagent (Invitrogen, Carlsbad, CA, USA). The synthesis of cDNA was performed by reverse transcription as reported [24].

### Real-time quantitative PCR

*KRAS* and *NRAS* expressions were detected by real-time quantitative PCR (RQ-PCR) using AceQ qPCR SYBR Green Master Mix (Vazyme Biotech Co., Piscataway, NJ, USA). The primers of *KRAS* and *NRAS* expressions were used as reported [25, 26]. PCR conditions were conducted at 95 °C for 5 min, followed by 40 cycles at 95 °C for 10 s, 60 °C for 30 s, 72 °C for 32 s, and 75 °C for 32 s. Housekeeping gene *ABL* was used to calculate the abundance of *KRAS* and *NRAS* mRNA. The detection of *ABL* expression was performed with primers as reported [27]. Both positive [K562 cell lines samples, cultured in RPMI 1640 medium containing 10% fetal calf serum (ExCell Bio, Shanghai, China)] and negative controls (ddH_2_O) were included in each assay. Relative *KRAS* and *NRAS* expressions levels were calculated using 2^-∆∆CT^ method.

### DNA extraction and gene mutation detection

Genomic DNA was isolated from BMMNCs using genomic DNA purification kit (Gentra, Minneapolis, MN, USA). The hot-spot mutations (codons 12, 13 and 61) of *KRAS* and *NRAS* were screened using high-resolution melting analysis (HRMA) as reported [8]. All positive samples were confirmed by DNA sequencing. The other gene mutations including *NPM1*, *C-KIT*, *DNMT3A*, *IDH1*, *IDH2*, and *U2AF1* were also detected by HRMA [28–33], whereas *FLT3*-ITD and *CEBPA* mutations were examined by DNA sequencing (BGI Tech Solutions Co., Shanghai, China) [34, 35].

### Gene Expression Omnibus datasets

Two independent cohorts of CN-AML patients (78 and 162 patients) from GEO data (http://www.ncbi.nlm.nih.gov/geo/; accession number GSE12417) were applied to analyze the prognostic impact of *KRAS* and *NRAS* expressions using the online web tool Genomicscape (http://genomicscape.com/microarray/survival.php) [36, 37].

### Statistical analyses

Mann-Whitney’s U test and Pearson Chi-square/Fisher exact test were employed to compare the difference of continuous and categorical variables between two groups. The relationship between *KRAS* expression and *NRAS* expression was analyzed by Spearman test. The impact of *KRAS* and *NRAS* expressions on prognosis was determined by Kaplan-Meier and Cox regression analyses. All the statistical analyses were performed through SPSS 20.0 software package. For all analyses, a two-tailed *P* value less than 0.05 was determined as statistically significant.

## Abbreviations

AML: acute myeloid leukemia
CR: complete remission
CN-AML: cytogenetically normal AML
GEO: Gene Expression Omnibus
MDS: myelodysplastic syndrome
BM: bone marrow
WHO: World Health Organization
BMMNCs: BM mononuclear cells
APL: acute promyelocytic leukemia
ATRA: all-trans retinoic acid
RQ-PCR: real-time quantitative PCR
HRMA: high-resolution melting analysis
OS: overall survival
